# Identification and Analysis of *PEPC* Gene Family Reveals Functional Diversification in Orchidaceae and the Regulation of Bacterial-Type *PEPC*

**DOI:** 10.3390/ijms25042055

**Published:** 2024-02-08

**Authors:** Ruyi Li, Xuyong Gao, Yuwei Wu, Chunyi Wei, Ming-He Li, Ding-Kun Liu, Zhong-Jian Liu

**Affiliations:** 1 Key Laboratory of National Forestry and Grassland Administration for Orchid Conservation and Utilization at Landscape Architecture and Arts, Fujian Agriculture and Forestry University, Fuzhou 350002, China; 1211775026@fafu.edu.cn (R.L.); 1201775011@fafu.edu.cn (X.G.); 1211775050@fafu.edu.cn (Y.W.); 1211775046@fafu.edu.cn (C.W.); fjalmh@fafu.edu.cn (M.-H.L.); 2 Fujian Colleges and Universities Engineering Research Institute of Conservation and Utilization of Natural Bioresources, Fujian Agriculture and Forestry University, Fuzhou 350002, China

**Keywords:** crassulacean acid metabolism (CAM), *PEPC* gene family, orchids, evolution, bacterial-type *PEPC*

## Abstract

Phosphoenolpyruvate carboxylase (PEPC) gene family plays a crucial role in both plant growth and response to abiotic stress. Approximately half of the Orchidaceae species are estimated to perform CAM pathway, and the availability of sequenced orchid genomes makes them ideal subjects for investigating the *PEPC* gene family in CAM plants. In this study, a total of 33 *PEPC* genes were identified across 15 orchids. Specifically, one *PEPC* gene was found in *Cymbidium goeringii* and *Platanthera guangdongensis*; two in *Apostasia shenzhenica*, *Dendrobium chrysotoxum*, *D. huoshanense*, *Gastrodia elata*, *G. menghaiensis*, *Phalaenopsis aphrodite*, *Ph. equestris*, and *Pl. zijinensis*; three in *C. ensifolium*, *C. sinense*, *D. catenatum*, *D. nobile*, and *Vanilla planifolia*. These *PEPC* genes were categorized into four subgroups, namely PEPC-i, PEPC-ii, and PEPC-iii (PTPC), and PEPC-iv (BTPC), supported by the comprehensive analyses of their physicochemical properties, motif, and gene structures. Remarkably, PEPC-iv contained a heretofore unreported orchid *PEPC* gene, identified as *VpPEPC4*. Differences in the number of *PEPC* homolog genes among these species were attributed to segmental duplication, whole-genome duplication (WGD), or gene loss events. *Cis*-elements identified in promoter regions were predominantly associated with light responsiveness, and circadian-related elements were observed in each PEPC-i and PEPC-ii gene. The expression levels of recruited BTPC, *VpPEPC4*, exhibited a lower expression level than other *VpPEPC*s in the tested tissues. The expression analyses and RT-qPCR results revealed diverse expression patterns in orchid *PEPC* genes. Duplicated genes exhibited distinct expression patterns, suggesting functional divergence. This study offered a comprehensive analysis to unveil the evolution and function of *PEPC* genes in Orchidaceae.

## 1. Introduction

Phosphoenolpyruvate carboxylase (PEPC) is a plant enzyme encoded by a small gene family, with each member exhibiting specialized functions [1,2,3]. It is widely distributed in various organisms, including plants, archaea, bacteria, cyanobacteria, green algae, and protozoa [4]. The *PEPC* gene can be further classified into plant-type PEPC (PTPC) and bacterial-type PEPC (BTPC) [5,6,7]. PTPC genes encoded 100–110 kDa polypeptides with 9–10 introns that contained a conserved phosphorylation site in the N-terminal, whereas BTPC genes encoded 116–118 kDa polypeptides with 19–21 introns [2,8]. *PEPC* genes are mainly involved in catalyzing HCO^3−^ and phosphoenolpyruvate (PEP) to produce oxaloacetate (OAA) and subsequent malate in the CAM pathway and C_4_ pathway [9,10]. In addition, *PEPC* also serves as a housekeeping gene, playing crucial physiological or metabolic roles in non-photosynthetic organisms, such as stomatal movements regulation [11], seed germination [12], fruit maturation [7], and the biosynthesis of oil and protein [13].

The *PEPC* gene family plays a vital role in both monocot and dicot species, and its characterization has been extensively studied in various plants, including *Arabidopsis thaliana* [8], *Saccharum* spp. [14], Oncidiinae spp. [3], *Kalanchoë* spp. [15], and other plants. Four *PEPC*s have been identified in *A. thaliana* with three plant-type phosphoenolpyruvate carboxylase (PTPC) and one bacterial-type phosphoenolpyruvate carboxylase (BTPC) [8], and ten in *Glycine max* with seven PTPC and three BTPC [16]. Five *PEPC*s were identified in sedges, with the recruitment of the *ppc-1* gene into the C_4_ pathway [17]. Comprehensive genomic and transcriptomic analyses indicated that a *PEPC* gene (*Dca1b*) might be associated with CAM in *Dendrobium catenatum*, as evidenced by its higher expression in major photosynthetic tissues [18]. Numerous functional studies of *PEPC* genes have also been reported. *Setaria viridis*, characterized by the low expression levels of *PEPC*, grows slowly even under high concentrations of CO_2_ [19]. The potential functions of *PEPC* were studied in response to abiotic stresses, such as salt, cold, and drought. The *cis*-regulatory elements related to various abiotic stresses were identified in the promoter regions of *PEPC* in soybean, and their transcript abundance and enzyme activities were altered by aluminum, cold, salt, and other stress [16]. Under the treatment of 25 μmol AlCl_3_ (pH 4.3) for a duration of 24 h, the PEPC activity of soybean leaves increased slightly, while that of roots increased first and then decreased [16]. The overexpression of *PEPC* significantly enhanced photosynthetic efficiency and tolerance to environmental abiotic stresses [20,21,22].

Crassulacean acid metabolism (CAM) is one of the important carbon fixation pathways due to its higher water-use efficiency and drought tolerance [9,23]. PEPC, the key enzyme for the CAM pathway at night, fascinated the researchers. Based on the transcriptomic dataset, the *PEPC* gene family has been identified in Oncidiinae, including C_3_, weak-CAM, and strong-CAM plants, and the number of *PEPCs* increased gradually along with the type of photosynthesis [3]. The *PEPC*s have also been investigated based on genomic analyses, such as three *PEPC*s in pineapple [24] and two in *Cymbidium mannii* [25]. The diel expression patterns of *PEPC* genes have been reported in many CAM plants [24,25,26,27]. In *C. mannii*, a copy of the *PEPC* gene (*PPC1;3*) exhibited a markedly higher expression level than other copies and displayed rhythmic expression and day-night differences in protein abundance, implying that this copy played a dominant role in the fixation of CO_2_ [25]. Moreover, the transgenic *Kalanchoë laxiflora* with a loss of the *PEPC* gene significantly reduced the nocturnal CO_2_ fixation and malate accumulation, and perturbations in stomatal closure during the light period were observed [15].

CAM plants are widely distributed in over 400 genera from 37 families of higher plants, such as Bromeliaceae, Crassulaceae, and Orchidaceae [28,29]. Among these families, Orchidaceae showed the highest diversity of CAM plants, with an estimated half of the species performing CAM pathways [30,31]. In recent years, several orchid genomes and transcriptomes have been sequenced [25,32,33,34,35,36,37,38,39,40,41,42,43,44,45], providing an excellent opportunity to elucidate the evolution and molecular regulation mechanism of CAM. However, our understanding of the *PEPC* gene family in orchids remains limited. The majority of research on the orchid *PEPC* gene family has been primarily based on the transcriptome, and comprehensive analyses of the PEPC gene family were scarce, such as *cis*-acting prediction, motif analysis, and so on. In this study, we performed genome-wide identification and comparative and expression analyses of the *PEPC* gene family in 15 orchids, namely *Apostasia shenzhenica* [32], *C. ensifolium* [33], *C. goeringii* [34], *C. sinense* [35], *D. catenatum* [36], *D. chrysotoxum* [37], *D. huoshanense* [38], *D. nobile* [39], *Gastrodia elata* [40], *G. menghaiensis* [41], *Phalaenopsis aphrodite* [42], *Ph. equestris* [43], *Platanthera guangdongensis* [44], *Pl. zijinensis* [44], and *Vanilla planifolia* [45], to investigate the characteristics and function of orchid *PEPC* genes. Our results can help in understanding the evolution and function of the orchid *PEPC* gene family.

## 2. Results

### 2.1. Identification and Phylogenetic Analysis of PEPC Genes

In total, 33 *PEPC* genes were identified across 15 orchid genomes, and the number of *PEPC* genes ranged from one to three in different species (one in *C. goeringii* and *Pl. guangdongensis*; two in *A. shenzhenica*, *D. chrysotoxum*, *D. huoshanense*, *G. elata*, *G. menghaiensis*, *Ph. aphrodite*, *Ph. equestris*, and *Pl. zijinensis*; three in *C. ensifolium*, *C. sinense*, *D. catenatum*, *D. nobile*, and *V. planifolia*). The new names of the predicted *PEPC* genes are listed in Table 1. For a more detailed analysis of the 33 PEPCs, their physicochemical properties were predicted using ExPASy (https://web.expasy.org/protparam/, accessed on 8 August 2023). The results showed that all PEPCs shared similar physicochemical properties (Table 1). These PEPC sequences exhibited slight variation in the amino acid lengths, ranging from 834 aa (CsPEPC1) to 1,076 aa (CePEPC2), with an average length of 960 aa. The molecular weights (MW) varied from 95.42 kDa (CsPEPC1) to 122.43 kDa (CePEPC2), with an average MW of 109.48 kDa. The theoretical isoelectric point ranged from 5.71 (AsPEPC3 and PgPEPC3) to 6.64 (DePEPC1), with an average of 6.03. All PEPCs were regarded as acidic (theoretical isoelectric point < 7) and hydrophilic (grand average of hydropathicity < 0) proteins (Table 1). Subcellular localization prediction suggested that all PEPCs are likely only located in the cytoplasm.

**Figure 1:**
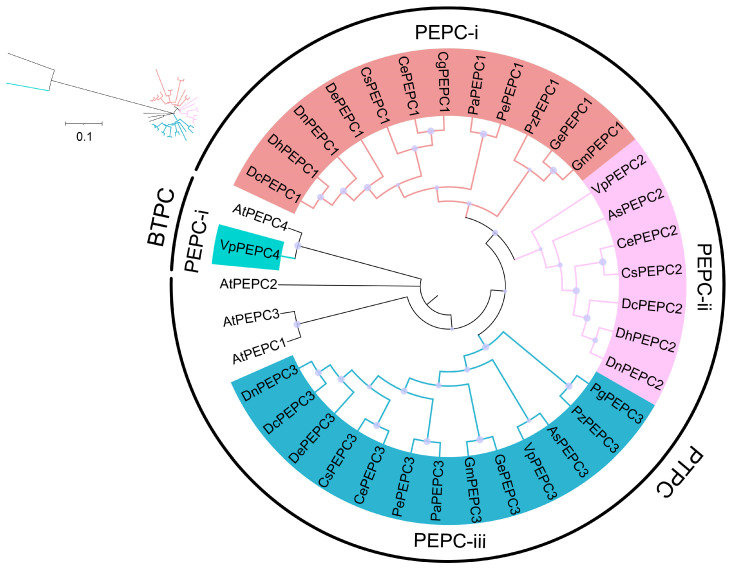
Phylogenetic trees of PEPC proteins based on 33 orchid PEPC proteins and 4 AtPEPC proteins. The different sizes of the circle on the nodes represent bootstrap percentages; the upper left corner shows the phylogenetic topology structure. PEPC-i, PEPC-ii, PEPC-iii, and PEPC-iv are grouped together as indicated in pale red, pink, blue, and green, respectively. *A. shenzhenica*, *C. ensifolium*, *C. goeringii*, *C. sinense, D. catenatum, D. chrysotoxum*, *D. huoshanense*, *D. nobile*, *G. elata*, *G. menghaiensis*, *Ph aphrodite*, *Ph. equestris*, *Pl guangdongensis*, *Pl. zijinensis*, and *V. planifolia* are labeled as As, Ce, Cg, Cs, Dc, De, Dh, Dn, Ge, Gm, Pa, Pe, Pg, Pz, and Vp, respectively.

To explore the evolution of *PEPC* genes among the 15 orchid genomes, phylogenetic trees were constructed using the neighbor-joining (NJ) method. The multiple protein sequence alignment of the 1297 aa PEPC fragments from 33 PEPC proteins showed 346 conserved sites, 698 variable sites, 281 parsimony-informative sites, and 364 singleton sites. The analysis demonstrated that the 33 orchid PEPC proteins are single origin and can be divided into PTPC and BTPC, with 32 and 1 members in the orchids, respectively (Figure 1). BTPC showed a distant branch with PTPC. Further classification of 33 PEPCs was divided into four subgroups: PEPC-i, PEPC-ii and PEPC-iii (PTPC), and PEPC-iv (BTPC). PEPC-iii (13 members) contained the most complete PEPC members of 15 orchids, followed by PEPC-i (12 members). Notably, this study revealed that PEPC-iv contained an orchid PEPC from *V. planifolia* (VpPEPC4), which has not been reported before.

### 2.2. Motif and Gene Structure Analysis of PEPC Genes

**Figure 2:**
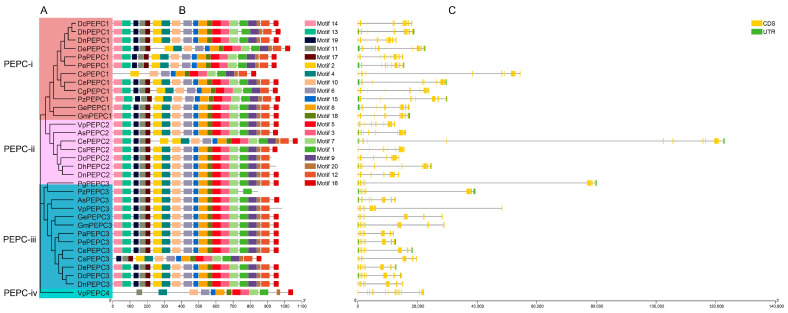
Gene structure, conserved motifs, and domains of *PEPC*s. (**A**) The NJ tree contains 33 orchid *PEPC*s. (**B**) Squares of different colors represent conserved motifs of *PEPC*s. (**C**) Squares of different colors represent the gene structures of *PEPC*s.

In this study, a detailed analysis of PEPC proteins was conducted using the MEME program to identify motif patterns, with a predefined upper limit of 20 motifs (Figure 2B). The proteins encoded by the PTPC were conserved and similar in motif patterns, with the number of PEPC motifs ranging from 14 to 21. The protein encoded by BTPC had 13 motifs. Both PTPC and BTPC shared an identical motif pattern partly, specifically the order of motifs 10, 6, 15, 8, 18, 5, 3, 7, and 1. However, some differences between the two types were observed. Motifs 13, 19, and 2 were only presented in PTPC. The number of BTPC motifs was lower than that in PTPC, indicating a discernible divergence between the two types.

To obtain deeper insights into the potential structural evolution of *PEPC*s, we conducted a comparative analysis of their exon–intron composition (Figure 2C). Different exon–intron structures were observed between the two types, but no discernible differences were observed within clades. PTPC consisted of 9–12 exons and 8–11 introns, whereas BTPC exhibited a more complex structure with 20 exons and 19 introns. While a similarity in the gene structure was found within each clade, orchid *PEPC*s demonstrated a substantial degree of variability in intron length and numbers in comparison with *A. thaliana*. Most orchid *PEPC*s exhibited longer intron than *A. thaliana.* Some orchid *PEPC*s exhibited variations in the number of introns; for instance, *CePEPC2* has 11 introns, whereas *CsPEPC1*, *CsPEPC3*, and *PzPEPC3* have only eight introns.

### 2.3. Cis-Elements in the Promoter Regions of PEPC Genes

**Figure 3:**
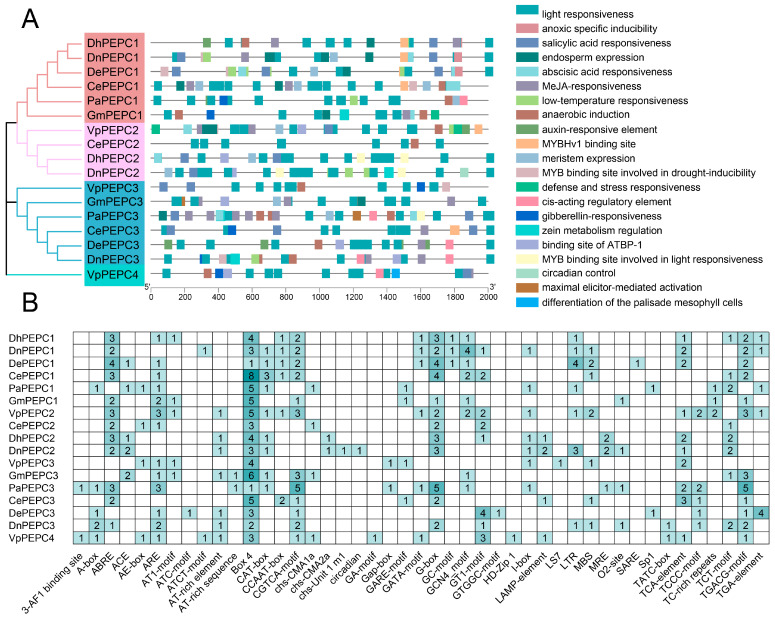
*Cis*-acting elements in the promoter regions of *PEPC* genes. (**A**) Functions of *cis*-acting elements in different orchid *PEPC*s. (**B**) Number of *cis*-acting elements in different orchid *PEPC*s.

To investigate the regulatory functions of the orchid *PEPC*s, we retrieved the 2000 bp promoter regions of six orchids to identify putative *cis*-acting regulatory elements (CREs). A comprehensive analysis unveiled a total of 402 CREs, including 45 types and 21 responsive functions (Figure 3 and Supplementary Table S1). *Cis*-element functions included developmental elements such as light responsiveness, endosperm expression, meristem expression, and circadian control; phytohormone responsiveness for abscisic acid (ABA), auxin, gibberellin, methyl jasmonate (MeJA), and salicylic acid; stress responsiveness such as anoxic and low-temperature (Figure 3A). Each *PEPC* gene harbored multiple types of elements, with light responsiveness (186, 46.3%) emerging as the most prevalent functional category. MeJA-responsiveness element (50, 12.4%) ranked as the second most abundant, followed by abscisic acid responsiveness (30, 7.5%). Among these elements, Box4 comprised the most common elements (64, 15.9%) present in each *PEPC*, followed by G-Box (33, 8.2%) (Figure 3B). Elements such as ABRE, G-box, and circadian elements were closely associated with the regulation of genes involved in the circadian rhythm. In this study, ABRE and G-box elements were present in each *PEPC* gene at PEPC-i and PEPC-ii, except for *PaPEPC1*. In contrast, the circadian element was only present in *DnPEPC2*.

### 2.4. Chromosomal Localization and Collinearity Analysis of PEPC Genes

**Figure 4:**
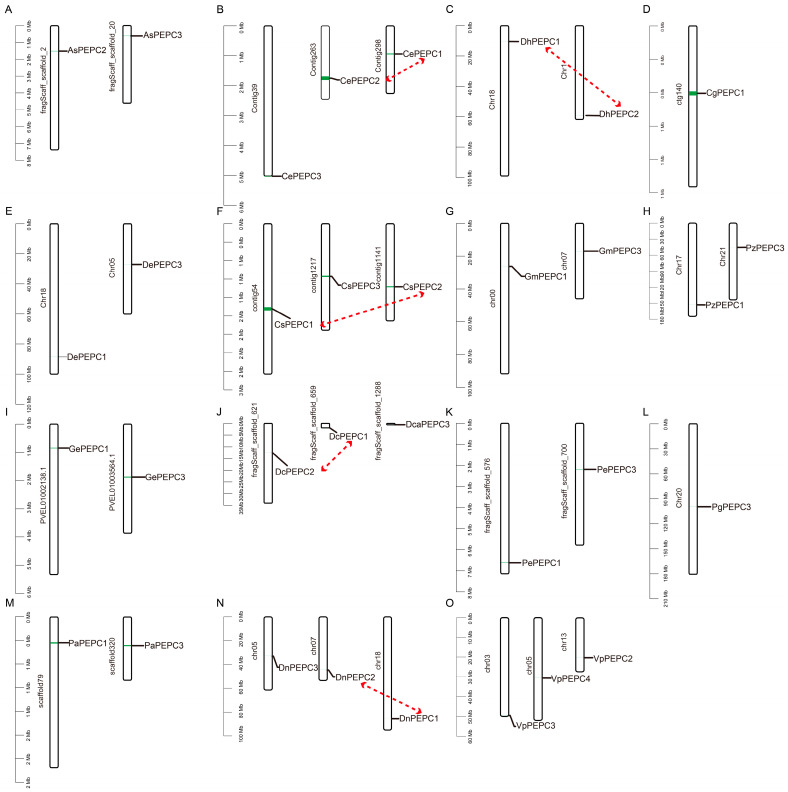
Chromosomal localization of *PEPC* genes. The scale bars on the left are the length (Mb) of the chromosomes of each orchid. Red lines connect the possible duplicated genes. (**A**) *A. shenzhenica*; (**B**) *C. ensifolium*; (**C**) *D. huoshanense*; (**D**) *C. goeringii*; (**E**) *D. chrysotoxum*; (**F**) *C. sinense*; (**G**) *G. menghaiensis*; (**H**) *Pl. zijinensis*; (**I**) *G. elata*; (**J**) *D. catenatum*; (**K**) *Ph. equestris*; (**L**) *Pl. guangdongensis*; (**M**) *Ph. aphrodite*; (**N**) *D. nobile*; (**O**) *V. planifolia*.

We conducted gene localization on chromosomes and gene duplication analysis to elucidate the homologous relationships among genes across the 15 orchid species. Each *PEPC* gene was situated on distinct chromosomes within all species (Figure 4). A total of five gene pairs were identified within species (*CePEPC1* and *CePEPC2*, *CsPEPC1* and *CsPEPC2*, *DcPEPC1* and *DcPEPC2*, *DhPEPC1* and *DhPEPC2*, and *DnPEPC1* and *DnPEPC2*), and were regarded as duplicated genes. The scattered distribution of *PEPC* genes on the chromosomes indicated that the duplicated genes in each orchid may result from segmental duplication.

**Figure 5:**
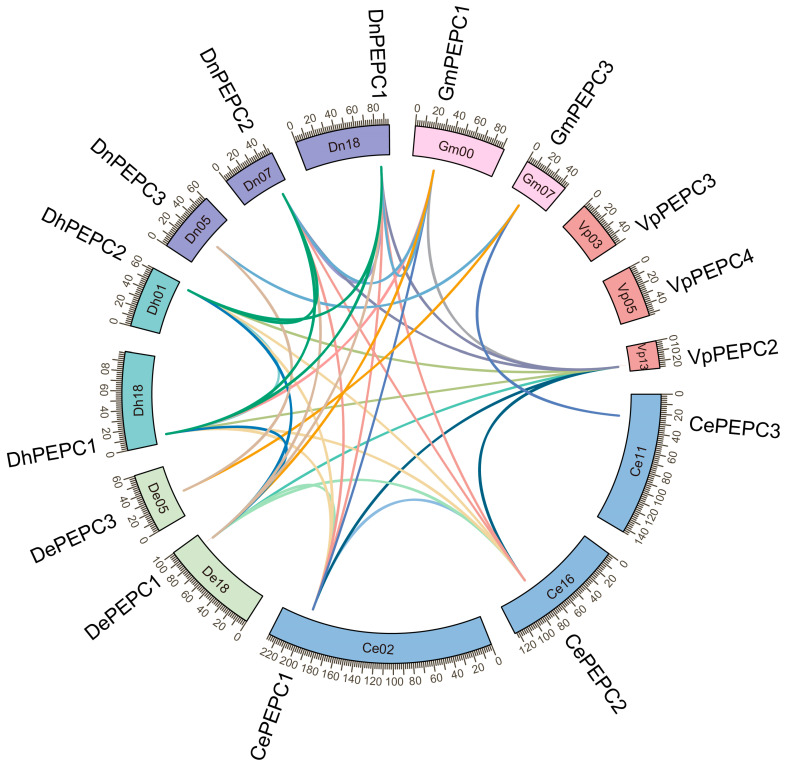
Location and orthologs or paralogs of 38 *PEPC* gene pairs intra- or inter-species of *G. menghaiensis*, *V. planifolia*, *C. ensifolium*, *D. chrysotoxum*, *D. huoshanense*, and *D. nobile* genomes. The chromosomes of *G. menghaiensis*, *V. planifolia*, *C. ensifolium*, *D. chrysotoxum*, *D. huoshanense*, and *D. nobile* were shown with different colors and labeled as Gm, Vp, Ce, De, Dh, and Dn, respectively. The gene pairs among different species are shown with different colors.

To study the evolutionary regulation of the orchid *PEPC* gene family, MCScanX was used to identify duplicated gene pairs. The collinear relationships among the 15 *PEPC* genes of *C. ensifolium*, *D. chrysotoxum*, *D. huoshanense*, *D. nobile*, *G. menghaiensis*, and *V. planifolia* were examined (Figure 5). Duplicated genes intra- or inter-species were investigated, and 38 gene pairs were obtained. To explore the different selective constraints on duplicated *PEPC* genes in six orchids, the 38 gene pairs were selected for calculating the ratio of the number of non-synonymous substitutions per non-synonymous site (*Ka*) to the number of synonymous sites (*Ks*). The results showed that the *Ka*/*Ks* ratios of all *PEPC* gene pairs were less than 1, implying that the orchid *PEPC* genes mainly experienced strong purifying selection after segmental duplication or WGD (Supplementary Table S2).

### 2.5. Expression Analysis of PEPC Genes

**Figure 6:**
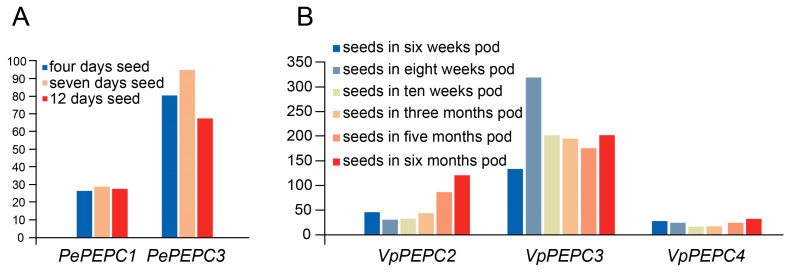
Temporal expression patterns of *PEPC* genes in orchids. Gene expression levels were quantified in FPKM. (**A**) *Ph. equestris*; (**B**) *V. planifolia*.

To elucidate the function of *PEPC* genes in orchids, the spatial and temporal expression patterns of *PEPC* genes in orchids were conducted based on transcriptome datasets. Here, we focused on the developmental seed transcriptome of *Ph. equestris*, spanning the 4, 7, and 12 days (Figure 6A). The temporal analysis unveiled dynamic expression patterns; the expression of *PePEPC*s was upregulated from the fourth day to the seventh day, followed by a subsequent downregulation on the 12th day. Notably, *PePEPC3* consistently exhibited higher expression levels than *PePEPC1*. In addition, the developmental seed expression of *V. planifolia* within six, eight, and ten weeks, as well as three, five, and six-month pods were investigated based on the transcriptome data (Figure 6B). Distinct expression patterns of *VpPEPC*s in seeds were demonstrated. *VpPEPC3* was most abundantly expressed, while *VpPEPC4*, a BTPC, was barely expressed. *VpPEPC3* demonstrated an upward trend from six weeks to eight weeks, followed by a subsequent decline, and a slight rise in the six months. These results collectively underscored the intricate dynamics of *PEPC* gene expression and emphasized their important roles in seed germination.

**Figure 7:**
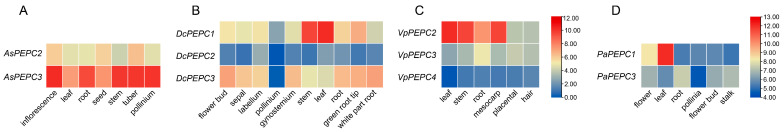
Spatial expression patterns of *PEPC* genes in orchids. Gene expression levels (FPKM) were normalized by log2 scale. (**A**) *A. shenzhenica*; (**B**) *D. catenatum*; (**C**) *V. planifolia*, mesocarp, placental, and hairs in three months pod; (**D**) *Ph. aphrodite*, flower bud: 2.5 cm, stalk: 1.5–3 cm.

The spatial expression patterns of *PEPC* genes in *A. shenzhenica*, *D. catenatum*, *Ph aphrodite*, *and V. planifolia* were analyzed to investigate gene biological functions and functional diversity (Figure 7). The expression results revealed that the *PEPC* gene exhibited widespread expressions in both vegetative tissues (leaf, root, and stem) and reproductive tissues (flower, pollinium, and seed). These findings indicated a diverse array of biological functions in growth and development associated with *PEPC* genes. Various expression patterns were observed for *PEPC* genes in different tissues. In *A. shenzhenica*, *AsPEPC3* displayed prominent expressions in sequenced tissues, while *AsPEPC2* exhibited lower expressions in all tissues (Figure 7A). The pronounced expression of *AsPEPC3* in floral tissues (inflorescence and pollinium) and vegetative tissues (root, stem, and tuber), suggested its involvement in multiple aspects of plant growth. In *D. catenatum*, *DcPEPC1* exhibited the highest expression levels in the stem and leaf, while *DcPEPC2* showed minimal expressions (Figure 7B), indicating the function divergence between duplicated *PEPC* genes. *DcPEPC3* exhibited high expressions in all the tested tissues, but lower than the expression level of *DcPEPC1* in the stem and leaf. In *V. planifolia*, the bacterial-type *PEPC* (*VpPEPC4*), not reported before, exhibited lower expressions than other *VpPEPC*s in all the tested tissues (Figure 7C). *VpPEPC2* displayed the highest expression level in the leaf, stem, root, and mesocarp of the pod. In *Ph aphrodite*, *PaPEPC1* showed the highest expression in the leaf, followed by the flower, and lowest in the root, pollinia, flower bud, and stalk (Figure 7D). *PaPEPC3* demonstrated a relatively low expression level in tested tissues. In summary, orchid *PEPC* genes showed key functions in various tissues, including seed germination, photosynthetic function, floral development, and root elongation.

### 2.6. RT-qPCR of PEPC Genes

**Figure 8:**
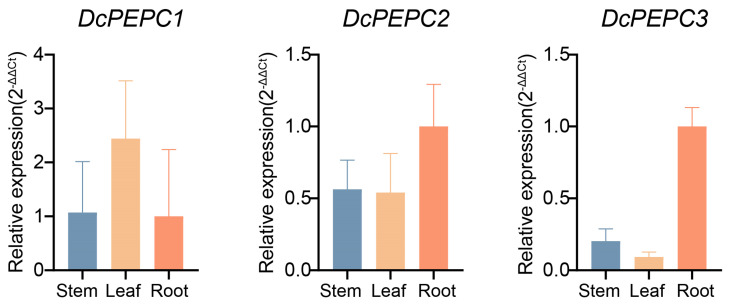
RT-qPCR analysis of three *DcPEPC*s genes in *D. catenatum* in different tissues (stem, leaf, and root). Error bars indicate the SD of three biological replicates.

RT-qPCR was employed to corroborate the expression profiles of three *PEPC* genes (*DcPEPC1*, *DcPEPC2*, and *DcPEPC3*) in the stem, leaf, and root tissues. The results showed that *DcPEPC1* exhibited high expressions in the leaf, followed by the stem and root (Figure 8, Supplementary Table S5). *DcPEPC3* exhibited the highest expression levels in the root, followed by the stem and leaf. These results aligned with the expression patterns observed in the transcriptome data. However, a slight difference in the expression pattern of *DePEPC2* was observed between RT-qPCR and transcriptome data. In RT-qPCR, *DePEPC2* demonstrated the highest expression level in the root, while transcriptome data indicated a higher expression in the leaf. The expression profiles of three genes in three tissues, as determined by RT-qPCR, were largely consistent with the transcriptome data, substantiating the accuracy of the aforementioned expression patterns.

## 3. Discussion

### 3.1. Identification and Phylogenetic Analysis of PEPC Genes

The enzymes encoded by *PEPC* genes are responsible for primary CO_2_ fixation into OAA and malate [46]. The *PEPC* gene family plays a vital role in plant growth and development and has been studied in several plant families, including Crassulaceae, Fabaceae, and Poaceae [16,26,47]. However, the systematic identification or functional reports of the *PEPC* gene family in Orchidaceae based on the genome data remain limited. This study identified 33 *PEPC* genes from 15 orchid genomes that contained both C_3_ and CAM plants [48,49,50,51] (Table 1). The results showed that each species contained one to three *PEPC* homolog genes, fewer than the counts found in *Gossypium* [52], wheat, and sorghum [10]. In contrast to the varying tendency of *PEPC* numbers in Oncidiinae [3], the quantity of *PEPC* appeared to be unrelated to the type of photosynthesis in this study. This suggested that the gene dosage effect played a minor role in the CAM pathway, consistent with other perspectives [18,43].

A total of 33 PEPC proteins from 15 orchid genomes and four PEPC proteins from *A. thaliana* were combined to construct a phylogenetic tree. The result showed that orchid PEPCs were divided into two subfamilies: PTPC and BTPC. Further classification of the PTPC revealed three subclades (Figure 1). The phylogenetic relationship was consistent with previous studies [3,18,52]. Both phylogenetic and sequence analyses converged to suggest a single origin prior to the divergence of bacteria and plant lineages (Figure 1, Table 1). *PEPC* genes with different coding types in plants evolved independently and showed distant relationships (Figure 1). PEPC-iii contained 13 *PEPC* members, including most orchid species, followed by PEPC-i, implying that compared with PEPC-ii and PEPC-iv, the functional characteristics of *PEPC* in these two subgroups were relatively conserved. The results indicated numerous duplication and loss events during the evolutionary trajectory, contributing to the variations in the numbers of *PEPC* homolog genes among different species [18].

In plants, the *PEPC* gene family mainly consists of PTPC and BTPC. Most species always recruit PTPC, whereas some species recruit BTPC. However, these genes were usually expressed at low levels or exhibited higher expression levels in non-photosynthetic tissues [53]. Notably, *Isoetes taiwanensis* has been documented to recruit BTPC, exhibiting expression levels surpassing those of PTPC [27]. This study highlighted the presence of *VpPEPC4* within the PEPC-iv (Figure 1), a novel finding yet to be reported in orchids. However, conclusive evidence regarding the functional specialization of this *PEPC* gene within CAM in *V. planifolia* requires validation through further transcriptomic studies.

### 3.2. Motif and Gene Structure Analysis of PEPC Genes

The motif and domain analysis revealed the high conservation of *PEPC* genes in orchids (Figure 2), consistent with other species, such as maize and sorghum [10]. The gene structure analysis revealed that the number of introns and exons in most orchid *PEPC* genes was similar to *AtPEPC*s but with some slight variations in the intron length. The gene family of orchids exhibited a longer intron length than the homolog genes in *A. thaliana*, which was reported previously [36,54]. The prevalence of longer introns in Orchidaceae likely represented a distinctive feature of this plant family. *CePEPC2* and *PgPEPC3* had unusually long introns (Figure 2C), suggesting that extremely long intron length might be attributed to species-specific evolution. The preference for longer intron over shorter counterparts was posited to release Hill–Robertson (HR) interference to enhance the efficiency of natural selection [55], which might be responsible for the biodiversity of Orchidaceae.

### 3.3. Cis-Elements in the Promoter Regions of PEPC Genes

Gene expression levels tend to be regulated by the *cis*-elements of promoter regions [56]. In this study, we identified the *cis*-elements within the 2000 bp promoter region of 17 *PEPC* genes from seven orchids (Figure 3). Our results showed a multitude of *cis*-elements implicated in light responsiveness, indicating the important role of light as a regulatory factor influencing *PEPC* gene functions. Many elements interacting with MYB transcription factors were identified. These MYB-binding sites were implicated in response to light and drought, suggesting the potential involvement of MYB transcription factors in regulating *PEPC* expression levels under stress conditions. Similar findings were found in C_4_ species [10]. This study suggested that the expression profile of the *PEPC* gene was regulated by multiple factors. Furthermore, compared with PEPC-iii, the promoter regions of *PEPC* genes at PEPC-i and PEPC-ii contained *cis*-elements associated with the circadian rhythm, implying that these two subgroups might be involved in photosynthesis.

### 3.4. Chromosomal Localization and Collinearity Analysis of PEPC Genes

Gene duplication events are a crucial factor in gene expansion or structural and functional divergence [57]. Using collinearity analysis, 38 gene pairs were identified (Figure 5), potentially attributed to WGD or segmental duplication, as evidenced by their chromosomal location [18,52]. The multiplicity of copies among different species ranged from one to three, indicative of probable duplication and loss events during the evolution [18]. Subsequently, these gene pairs were used for *Ka*/*Ks* analysis. The *Ka/Ks* ratio is essential for exploring genomic evolution [58], which can serve as an indicator of purifying selection (*Ka/Ks* < 1), neutral mutation (*Ka/Ks* = 1), or positive selection (*Ka/Ks* > 1). Purifying selection is recognized for its role in preserving existing biological functions [59]. Our study found that all the examined gene pairs exhibited *Ka/Ks* ratios less than 1 (Supplementary Table S2), suggesting that these *PEPC*s underwent exceedingly purifying selection, contributing to their high conservation in orchids.

### 3.5. Expression Analysis and RT-qPCR of PEPC Genes

During the extensive evolutionary history of orchid *PEPC* genes, duplicated genes may experience functional divergence [60]. Some *PEPC* genes in PEPC-i and PEPC-ii were identified as duplicated genes (Figure 1 and Figure 4). In *D. catenatum*, *DcPEPC1* demonstrated elevated expression levels, while its duplicated gene *DcPEPC2* exhibited minimal expression in all sequenced tissues (Figure 7B). The distinct expression patterns observed between the two genes suggested that the duplicated genes may experience neofunctionalization [52,60].

In *V. planifolia*, *VpPEPC4* was considered a bacterial-type *PEPC* gene based on phylogenetic analysis (Figure 1). However, the transcriptome data analysis showed that *VpPEPC4* was barely expressed in all the tested tissues (Figure 7C), contrasting with the high expression of BTPC in the green tissues of *I. taiwanensis* [27]. *VpPEPC2* exhibited elevated expression in the stem and leaves. In *D. catenatum*, *DcPEPC1* displayed prominent expression in the stem and leaf, much higher than that in other tissues (Figure 7B). The RT-qPCR experiments of *DcPEPC1* further verified these results (Figure 8). In *Ph. aphrodite*, *PaPEPC1* showed the most abundant expression level in the stem (Figure 7D). The expression patterns suggested the potential recruitment of *DcPEPC1, PaPEPC1*, and *VpPEPC2* in the CAM pathway. Therefore, combined with the prediction of *cis*-elements, we hypothesized that genes within the PEPC-i and PEPC-ii clade were CAM-related genes, supporting the previous report [18].

## 4. Materials and Methods

### 4.1. Data Sources

The *Arabidopsis thaliana* and predicted proteins were retrieved from the TAIR10 Genome Release (http://www.arabidopsis.org, accessed on 8 August 2022). The genome datasets of *A. shenzhenica* (accession number: PRJNA310678), *C. goeringii* (accession number: PRJNA749652), *C. sinense* (accession number: PRJNA743748), *D. catenatum* (accession number: PRJNA262478), *D. chrysotoxum* (accession number: PRJNA664445), *D. huoshanense* (accession number: PRJNA597621), *D. nobile* (accession number: PRJNA725550), *G. elata* (accession number: PRJNA386713), *G. menghaiensis* (accession number: PRJNA695369), *Ph. equestris* (accession number: PRJNA53913), *Pl. guangdongensis* and *Pl. zijinensis* (accession number: PRJNA739531), and *V. planifolia* (accession number: PRJNA633886) were obtained from the National Center for Biotechnology Information (NCBI); data for *Ph. aphrodite* was downloaded from orchidstra2 (http://orchidstra2.abrc.sinica.edu.tw/orchidstra2/pagenome.php, accessed on 7 January 2022); and data for *C. ensifolium* (accession number: PRJCA005355) was downloaded from the National Genomics Data Center (NGDC). The *PEPC* genes and predicted proteins were retrieved from the complete genomes. The transcriptomes of *A. shenzhenica*, *D. catenatum,* and *Ph. equestris* were obtained from Orchidbase (http://orchidbase.itps.ncku.edu.tw/est/home2012.aspx, accessed on 17 August 2023) [61]. The transcriptomes of *Ph. aphrodite* and *V. planifolia* were obtained from orchidstra2 (http://orchidstra2.abrc.sinica.edu.tw/orchidstra2/pagenome.php, accessed on 10 January 2024) [62].

### 4.2. Homolog Gene Identification and Sequence Analysis

The PEPC protein sequences of the model plant *A. thaliana* were used to conduct a blast search for orthologs within orchids based on the genomic data, employing the BLAST module (Blastp) of TBtools (version 1.121) [63], hitting with e-values less than 1 × 10^−10^. The detailed information of BLAST result was provided in Supplementary Table S3. Subsequently, all protein sequences further underwent validation using the NCBI batch CD-search tool (https://www.ncbi.nlm.nih.gov/Structure/bwrpsb/bwrpsb.cgi, accessed on 7 October 2023) with a threshold of 0.01 to scrutinize the conserved domain (PF00311), and proteins lacking the complete domain were eliminated. The MEME Suite 5.5.1 online tool (https://meme-suite.org/meme/, accessed on 7 October 2023) [64] was used to analyze motif composition. The intron–exon structure was discerned by the Visualize Gene Structure program of TBtools (version 1.121) [63]. Finally, the visualization of these results was accomplished using the Gene Structure View module of TBtools (version 1.121) [63].

The physicochemical properties of the protein sequences of PEPCs were predicted by ExPASy (https://web.expasy.org/protparam/, accessed on 8 August 2023) [65]. Additionally, subcellular localization predictions were performed on Plant-mPLoc (version 1.0) (http://www.csbio.sjtu.edu.cn/bioinf/plant-multi/, accessed on 8 August 2023) [66].

### 4.3. Multiple Sequence Alignment and Phylogenetic Tree Construction

Multiple sequences were aligned using MUSCLE integrated into MEGA 7 with the default parameters [67]. The phylogenetic tree of PEPCs was constructed using the neighbor-joining (NJ) method in MEGA7 [67]. Parameters included the ‘Poisson model’ and the ‘Pairwise deletion’ option with a bootstrap test of 1000 replicates. Finally, the phylogenetic tree was imported into iTOL (https://itol.embl.de/itol.cgi, accessed on 30 November 2023) [68] for refinement and polishing, employing circle and none-leaf sorting parameters. And Adobe Illustrator CC 2018 was used for supplementing clade labels.

### 4.4. Prediction of Cis-Acting Elements

The 2000 bp regions upstream of all *PEPC*s were extracted using Gtf/Gff3 Sequence Extract and Fasta Extract programs integrated into TBtools (version 1.121) [63]. The online website PlantCARE (https://bioinformatics.psb.ugent.be/webtools/plantcare/html/, accessed on 18 October 2023) [69] was utilized for identifying the putative *cis*-acting elements in the promoter regions. The Basic Biosequence View module of TBtools (version 1.121) [63] was used to display the findings of *cis*-acting element annotation and counts.

### 4.5. Chromosomal Localization and Collinearity Analysis

After obtaining information on the chromosomal locations of the *PEPC* gene family across 15 orchids from the corresponding genome annotations, the chromosomal location map was generated using the Gene Location Visualize program in TBtools (version 1.121) [63]. The collinear relationship among six orchids was delineated and visualized by the One Step MCScanX fast program of TBtools (version 1.121) [63]. Subsequently, the *Ka*/*Ks* ratios of duplicated genes were calculated by the Simple Ka/Ks Calculator integrated into TBtools (version 1.121) [63], using the Nei and Gojobori (NG) method. If Ka > Ks or Ka/Ks > 1, the gene was generally considered to undergo positive selection; if Ka = Ks or Ka/Ks = 1, the gene was subject to neutral evolution; and if Ka < Ks or Ka/Ks < 1, the gene underwent purifying selection [70].

### 4.6. Expression Patterns Analysis

The expression levels of *PEPC*s in *A. shenzhenica*, *D. catenatum*, and *Ph. equestris* were obtained from Orchidbase (http://orchidbase.itps.ncku.edu.tw/est/home2012.aspx, accessed on 17 August 2023) [61], *Ph. aphrodite* and *V. planifolia* were acquired from orchidstra2 (http://orchidstra2.abrc.sinica.edu.tw/orchidstra2/pagenome.php, accessed on 10 January 2024) [62]. The FPKM values of *PEPC*s were imported into the Heatmap program of TBtools (version 1.121) [63] for the generation of heat maps.

### 4.7. RT-qPCR

The plant materials used in this study were sourced from the National Orchid Germplasm Resources of Fujian Agriculture and Forestry University, Fuzhou, China. For RT-qPCR validation, three *PEPC* genes (*DcPEPC1*, *DcPEPC2*, and *DcPEPC3*) were selected. Total RNA extraction was carried out using the RaPure Total RNA Plus Kit (Magen Biotech Co., Ltd., Guangzhou, China), following the eighth method described in the kit manual for plant samples. Subsequently, cDNA synthesis was performed using the Hifair^®^ AdvanceFast 1st Strand cDNA Synthesis Kit (Yeasen Biotechnology, Shanghai, China). RT-qPCR analysis was conducted on the QuantStudio™ Real-Time PCR (Applied Biosystems, Waltham, MA, USA), employing the Hieff UNICON^®^ Universal Blue qPCR SYBR Green Master Mix (low rox) kit (Yeasen Biotechnology, Shanghai, China). The reaction system comprised a total volume of 20 µL, including 10 µL of Hieff UNICON Universal Blue qPCR SYBR Green Master Mix, 0.4 µL of forward primer (10 µM), 0.4 µL of reverse primer (10 µM), 2 µL of template DNA, and 7.2 µL of sterile ultrapure water. All experiments were conducted using three biological replicates and three technical replicates.

The Ct values obtained from RT-qPCR were analyzed using the 2^−ΔΔCT^ formula to determine the relative expression levels of the three genes in different tissues (stem, leaf, and root). The Ct values of roots were used as the control for calculation, and *DcGAPDH* was used as the internal reference for normalization. Detailed information on RT-qPCR primers was provided in Supplementary Table S4, and RT-qPCR data details were presented in Supplementary Table S5. The results of RT-qPCR were visualized using GraphPad Prism 8.0.1 for Windows (GraphPad Software version 8.0.1, San Diego, CA, USA, www.graphpad.com, accessed on 10 January 2024).

## 5. Conclusions

In this study, a comprehensive analysis of the orchid *PEPC* gene family was conducted. A total of 33 orchid *PEPC* genes were identified from 15 orchid species. Domain, motif pattern, and gene structure analyses revealed high conservation among all orchid *PEPC*. These genes were classified into two clades, PTBC and BTPC, exhibiting highly similar characteristics within each respective clade. PTPC was further classified into three subclades. Remarkably, an orchid *PEPC* in BTPC (*VpPEPC4*) was identified for the first time. However, the expression level of *VpPEPC4* was found to be lower than other *VpPEPC*s. The *cis-*acting element in light responsiveness acted in concert with the pivotal role of *PEPC*s in photosynthesis, and the circadian-related elements were found in most PEPC-i and PEPC-ii genes. The duplicated genes in *D. catenatum* showed distinct expression patterns and were validated using RT-qPCR results, indicating functional divergence in orchid evolution. These findings provided valuable insights into the characteristics and functions of *PEPC* in orchids.

## Figures and Tables

**Table 1:** Information of *PEPC* homologs in 15 orchid genomes and *A. thaliana*.

Gene Name ^a^	Gene ID	Intron	Amino Acid Number (aa)	Molecular Weight (kDa)	Theoretical Isoelectric Point	Grand Average of Hydrophobicity	Subcellular Localization
AsPEPC3	Ash006877	9	968	110.68	5.71	−0.405	Cytoplasm
AsPEPC2	Ash003090	9	961	109.32	6.28	−0.375	Cytoplasm
CePEPC3	JL001583	9	965	110.36	5.78	−0.379	Cytoplasm
CePEPC1	JL008709	9	964	109.90	6.04	−0.374	Cytoplasm
CePEPC2	JL007930	11	1076	122.43	6.33	−0.349	Cytoplasm
CgPEPC1	GL01041	10	960	109.57	6.30	−0.399	Cytoplasm
CsPEPC2	Mol008966	9	958	108.95	6.10	−0.336	Cytoplasm
CsPEPC3	Mol012516	8	865	99.01	6.17	−0.381	Cytoplasm
CsPEPC1	Mol013879	8	834	95.42	6.46	−0.354	Cytoplasm
DcPEPC3	Dca019608	9	965	110.14	5.81	−0.402	Cytoplasm
DcPEPC1	Dca010878	9	964	109.85	6.00	−0.390	Cytoplasm
DcPEPC2	Dca000344	9	920	105.12	5.85	−0.361	Cytoplasm
DePEPC3	Maker113806	9	965	110.21	5.76	−0.411	Cytoplasm
DePEPC1	Maker105367	10	1032	117.69	6.64	−0.328	Cytoplasm
DhPEPC1	Dhu000014644	9	976	111.33	5.98	−0.374	Cytoplasm
DhPEPC2	Dhu000025281	9	949	108.38	5.91	−0.368	Cytoplasm
DnPEPC3	cds-KAI0522578.1	9	965	110.22	5.75	−0.392	Cytoplasm
DnPEPC2	cds-KAI0516232.1	9	965	110.06	5.93	−0.356	Cytoplasm
DnPEPC1	cds-KAI0492914.1	9	965	110.04	6.16	−0.399	Cytoplasm
GePEPC1	Gel007954	9	965	109.94	6.04	−0.375	Cytoplasm
GePEPC3	Gel011478	9	965	110.23	6.05	−0.385	Cytoplasm
GmPEPC3	Gme.evm.model.scaffold_67.219	9	965	110.34	6.12	−0.396	Cytoplasm
GmPEPC1	Gme.evm.model.scaffold_80.68	9	965	109.89	6.02	−0.361	Cytoplasm
PaPEPC3	PAXXG242960-mRNA1	9	965	109.91	5.82	−0.376	Cytoplasm
PaPEPC1	PAXXG121890-mRNA1	9	953	108.39	5.97	−0.371	Cytoplasm
PePEPC3	Peq006904	9	965	110.03	5.82	−0.377	Cytoplasm
PePEPC1	Peq005747	9	953	108.35	6.00	−0.356	Cytoplasm
PgPEPC3	PGU005496	9	965	109.90	5.71	−0.361	Cytoplasm
PzPEPC1	PZI019529	9	974	111.01	5.91	−0.348	Cytoplasm
PzPEPC3	PZI023524	8	842	95.59	5.95	−0.359	Cytoplasm
VpPEPC2	Vpla_KAG0455466.1	9	965	109.76	6.02	−0.358	Cytoplasm
VpPEPC3	Vpla_KAG0489408.1	10	982	112.66	6.25	−0.433	Cytoplasm
VpPEPC4	Vpla_KAG0480734.1	19	1049	118.09	6.23	−0.350	Cytoplasm
AtPEPC1	AT1G53310.1	9	967	110.29	5.72	−0.391	Cytoplasm
AtPEPC2	AT2G42600.1	9	963	109.75	5.57	−0.387	Cytoplasm
AtPEPC3	AT3G14940.1	9	968	110.16	5.73	−0.389	Cytoplasm
AtPEPC4	AT1G68750.1	19	1032	116.59	6.68	−0.419	Cytoplasm

^a^ As, A. shenzhenica; Ce, C. ensifolium; Cg, C. goeringii; Cs, C. sinense; Dc, D. catenatum; De, D. chrysotoxum; Dh, D. huoshanense; Dn, D. nobile; Ge, G. elata; Gm, G. menghaiensis; Pa, Ph aphrodite; Pe, Ph. equestris; Pg, Pl guangdongensis; Pz, Pl. zijinensis; Vp, V. planifolia.

## Data Availability

All the data are provided within this manuscript.

## Supplementary Materials

The supporting information can be downloaded at: https://www.mdpi.com/article/10.3390/ijms25042055/s1.

## References

[1] Chollet R., Vidal J., O’Leary M.H. (1996). Phosphoenolpyruvate carboxylase: A ubiquitous, highly regulated enzyme in plants. Annu. Rev. Plant Biol..

[2] Izui K., Matsumura H., Furumoto T., Kai Y. (2004). Phosphoenolpyruvate carboxylase, a new era of structural biology. Annu. Rev. Plant Biol..

[3] Silvera K., Winter K., Rodriguez B.L., Albion R.L., Cushman J.C. (2014). Multiple isoforms of phosphoenolpyruvate carboxylase in the Orchidaceae (subtribe Oncidiinae), implications for the evolution of crassulacean acid metabolism. J. Exp. Bot..

[4] O’Leary B., Park J., Plaxton W.C. (2011). The remarkable diversity of plant *PEPC* (phosphoenolpyruvate carboxylase), recent insights into the physiological functions and post-translational controls of non-photosynthetic PEPCs. Biochem. J..

[5] Gennidakis S., Rao S., Greenham K., Uhrig R.G., O’Leary B., Snedden W.A., Lu C.F., Plaxton W.C. (2007). Bacterial- and plant-type phosphoenolpyruvate carboxylase polypeptides interact in the hetero-oligomeric Class-2 PEPC complex of developing castor oil seeds. Plant J..

[6] Igawa T., Fujiwara M., Tanaka I., Fukao Y., Yanagawa Y. (2010). Characterization of bacterial-type phosphoenolpyruvate carboxylase expressed in male gametophyte of higher plants. BMC Plant Biol..

[7] O’Leary B., Fedosejevs E.T., Hill A.T., Bettridge J., Park J., Rao S.K., Leach C.A., Plaxton W.C. (2011). Tissue-specific expression and post-translational modifications of plant-and bacterial-type phosphoenolpyruvate carboxylase isozymes of the castor oil plant, *Ricinus communis* L. J. Exp. Bot..

[8] Sánchez R., Cejudo F.J. (2003). Identification and expression analysis of a gene encoding a bacterial-type phosphoenolpyruvate carboxylase from *Arabidopsis* and rice. Plant Physiol..

[9] Borland A.M., Griffiths H., Hartwell J., Smith J.A.C. (2009). Exploiting the potential of plants with crassulacean acid metabolism for bioenergy production on marginal lands. J. Exp. Bot..

[10] Chen L., Yang Y., Zhao Z., Lu S., Lu Q., Cui C., Parry M.A.J., Hu Y.G. (2023). Genome-wide identification and comparative analyses of key genes involved in C_4_ photosynthesis in five main gramineous crops. Front. Plant Sci..

[11] Cousins A.B., Baroli I., Badger M.R., Ivakov A., Lea P.J., Leegood R.C., von Caemmerer S. (2007). The role of phosphoenolpyruvate carboxylase during C_4_ photosynthetic isotope exchange and stomatal conductance. Plant Physiol..

[12] Ruiz-Ballesta I., Baena G., Gandullo J., Wang L.Q., She Y.M., Plaxton W.C., Echevarría C. (2016). New insights into the post-translational modification of multiple phosphoenolpyruvate carboxylase isoenzymes by phosphorylation and monoubiquitination during sorghum seed development and germination. J. Exp. Bot..

[13] Singh J., Reddy G.M., Agarwal A., Chandrasekhar K., Sopory S.K., Reddy M.K., Kaul T. (2012). Molecular and structural analysis of C_4_-specific *PEPC* isoform from *Pennisetum glaucum* plays a role in stress adaptation. Gene.

[14] Besnard G., Pincon G., D’Hont A., Hoarau J.Y., Cadet F., Offmann B. (2003). Characterisation of the phosphoenolpyruvate carboxylase gene family in sugarcane (*Saccharum* spp.). Theor. Appl. Genet..

[15] Boxall S.F., Kadu N., Dever L.V., Kneřová J., Waller J.L., Gould P.J., Hartwell J. (2020). *Kalanchoë PPC1* is essential for crassulacean acid metabolism and the regulation of core circadian clock and guard cell signaling genes. Plant Cell.

[16] Wang N., Zhong X., Cong Y., Wang T., Yang S., Li Y., Gai J. (2016). Genome-wide analysis of phosphoenolpyruvate carboxylase gene family and their response to abiotic stresses in soybean. Sci. Rep..

[17] Besnard G., Muasya A.M., Russier F., Roalson E.H., Salamin N., Christin P.A. (2009). Phylogenomics of C_4_ photosynthesis in sedges (Cyperaceae), multiple appearances and genetic convergence. Mol. Biol. Evol..

[18] Deng H., Zhang L.S., Zhang G.Q., Zheng B.Q., Liu Z.J., Wang Y. (2016). Evolutionary history of *PEPC* genes in green plants, implications for the evolution of CAM in orchids. Mol. Phylogenet. Evol..

[19] Alonso-Cantabrana H., Cousins A.B., Danila F., Ryan T., Sharwood R.E., von Caemmerer S., Furbank R.T. (2018). Diffusion of CO_2_ across the mesophyll-bundle sheath cell interface in a C_4_ plant with genetically reduced PEP carboxylase activity. Plant Physiol..

[20] Tang Y., Li X., Lu W., Wei X., Zhang Q., Lv C., Song N. (2018). Enhanced photorespiration in transgenic rice over-expressing maize C_4_ phosphoenolpyruvate carboxylase gene contributes to alleviating low nitrogen stress. Plant Physiol. Biochem..

[21] Liu D., Hu R., Zhang J., Guo H.B., Cheng H., Li L., Borland A.M., Qin H., Chen J.-G., Muchero W. (2021). Overexpression of an agave phosphoenolpyruvate carboxylase improves plant growth and stress tolerance. Cells.

[22] Behera D., Swain A., Karmakar S., Dash M., Swain P., Baig M.J., Molla K.A. (2023). Overexpression of *Setaria italica* phosphoenolpyruvate carboxylase gene in rice positively impacts photosynthesis and agronomic traits. Plant Physiol. Biochem..

[23] Sunagawa H., Cushman J., Agarie S. (2010). Crassulacean acid metabolism may alleviate production of reactive oxygen species in a facultative CAM plant; the common ice plant *Mesembryanthemum crystallinum* L. Plant Prod. Sci..

[24] Ming R., VanBuren R., Wai C.M., Tang H., Schatz M.C., Bowers J.E., Lyons E., Wang M.L., Chen J., Biggers E. (2015). The pineapple genome and the evolution of CAM photosynthesis. Nat. Genet..

[25] Fan W., He Z.S., Zhe M., Feng J.Q., Zhang L., Huang Y.W., Liu F., Huang J.L., Ya J.D., Zhang S.B. (2023). High-quality *Cymbidium mannii* genome and multifaceted regulation of crassulacean acid metabolism in epiphytes. Plant Commun..

[26] Yang X., Hu R., Yin H., Jenkins J., Shu S., Tang H., Liu D.G., Weighill D.A., Yim W.C., Ha J. (2017). The *Kalanchoë* genome provides insights into convergent evolution and building blocks of crassulacean acid metabolism. Nat. Commun..

[27] Wickell D., Kuo L.Y., Yang H.P., Ashok A.D., Irisarri I., Dadras A., de Vries S., de Vries J., Huang Y.M., Li Z. (2021). Underwater CAM photosynthesis elucidated by *Isoetes* genome. Nat. Commun..

[28] Silvera K., Neubig K.M., Whitten W., Williams N.H., Winter K., Cushman J.C. (2010). Evolution along the crassulacean acid metabolism continuum. Funct. Plant Biol..

[29] Gamisch A., Winter K., Fischer G.A., Comes H.P. (2021). Evolution of crassulacean acid metabolism (CAM) as an escape from ecological niche conservatism in Malagasy *Bulbophyllum* (Orchidaceae). New Phytol..

[30] Smith J.A.C., Winter K. (1996). Crassulacean Acid Metabolism: Biochemistry, Ecophysiology and Evolution.

[31] Silvera K., Santiago L.S., Cushman J.C., Winter K. (2009). Crassulacean acid metabolism and epiphytism linked to adaptive radiations in the Orchidaceae. Plant Physiol..

[32] Zhang G.Q., Liu K.W., Li Z., Lohaus R., Hsiao Y.Y., Niu S.C., Wang J.Y., Lin Y.C., Xu Q., Chen L.J. (2017). The *Apostasia* genome and the evolution of orchids. Nature.

[33] Ai Y., Li Z., Sun W.-H., Chen J., Zhang D., Ma L., Zhang Q.-H., Chen M.-K., Zheng Q.-D., Liu J.-F. (2021). The *Cymbidium* genome reveals the evolution of unique morphological traits. Hortic. Res..

[34] Yang F., Gao J., Wei Y., Ren R., Zhang G., Lu C., Jin J., Ai Y., Wang Y., Chen L. (2021). The genome of *Cymbidium sinense* revealed the evolution of orchid traits. Plant Biotechnol. J..

[35] Sun Y., Chen G.-Z., Huang J., Liu D.-K., Xue F., Chen X.-L., Chen S.-Q., Liu C.-G., Liu H., Ma H. (2021). The Cymbidium goeringii genome provides insight into organ development and adaptive evolution in orchids. Ornam. Plant Res..

[36] Zhang G.Q., Xu Q., Bian C., Tsai W.C., Yeh C.M., Liu K.W., Yoshida K., Zhang L.S., Chang S.B., Chen F. (2016). The *Dendrobium catenatum* Lindl. genome sequence provides insights into polysaccharide synthase; floral development and adaptive evolution. Sci. Rep..

[37] Zhang Y., Zhang G.Q., Zhang D., Liu X.D., Xu X.Y., Sun W.H., Yu X., Zhu X., Wang Z.W., Zhao X. (2021). Chromosome-scale assembly of the *Dendrobium chrysotoxum* genome enhances the understanding of orchid evolution. Hortic. Res..

[38] Han B., Jing Y., Dai J., Zheng T., Gu F., Zhao Q., Zhu F., Song X., Deng H., Wei P. (2020). A chromosome-level genome assembly of Dendrobium huoshanense using long reads and Hi-C data. Genome Biol. Evol..

[39] Xu Q., Niu S.-C., Li K.-L., Zheng P.-J., Zhang X.-J., Jia Y., Liu Y., Niu Y.-X., Yu L.-H., Chen D.-F. (2022). Chromosome-scale assembly of the *Dendrobium nobile* genome provides insights into the molecular mechanism of the biosynthesis of the medicinal active ingredient of *Dendrobium*. Front. Genet..

[40] Yuan Y., Jin X., Liu J., Zhao X., Zhou J., Wang X., Wang D., Lai C., Xu W., Huang J. (2018). The *Gastrodia elata* genome provides insights into plant adaptation to heterotrophy. Nat. Commun..

[41] Jiang Y., Hu X.D., Yuan Y., Guo X.L., Chase M.W., Ge S., Li J.W., Fu J.L., Li K., Hao M. (2022). The *Gastrodia menghaiensis* (Orchidaceae) genome provides new insights of orchid mycorrhizal interactions. BMC Plant Biol..

[42] Chao Y.T., Chen W.C., Chen C.Y., Ho H.Y., Yeh C.H., Kuo Y.T., Su C.L., Yen S.H., Hsueh H.Y., Yeh J.H. (2018). Chromosome-level assembly; genetic and physical mapping of *Phalaenopsis aphrodite* genome provides new insights into species adaptation and resources for orchid breeding. Plant Biotechnol..

[43] Cai J., Liu X., Vanneste K., Tsai W.C., Liu K.W., Chen L.J., He Y., Xu Q., Bian C., Zheng Z. (2015). The genome sequence of the orchid *Phalaenopsis equestris*. Nat. Genet..

[44] Li M.H., Liu K.W., Li Z., Lu H.C., Ye Q.L., Zhang D., Wang J.Y., Li Y.F., Zhong Z.M., Liu X. (2022). Genomes of leafy and leafless *Platanthera* orchids illuminate the evolution of mycoheterotrophy. Nat. Plants.

[45] Hasing T., Tang H., Brym M., Khazi F., Huang T., Chambers A.H. (2020). A phased *Vanilla planifolia* genome enables genetic improvement of flavour and production. Nat. Food.

[46] Masumoto C., Miyazawa S.I., Ohkawa H., Fukudaa T., Taniguchia Y., Murayamaa S., Kusanob M., Saitob K., Fukayamaa H., Miyao M. (2010). Phosphoenolpyruvate carboxylase intrinsically located in the chloroplast of rice plays a crucial role in ammonium assimilation. Proc. Natl. Acad. Sci. USA.

[47] Huang W., Zhang L., Columbus J.T., Hu Y., Zhao Y., Tang L., Guo Z.H., Chen W.L., McKain M., Bartlett M. (2022). A well-supported nuclear phylogeny of Poaceae and implications for the evolution of C_4_ photosynthesis. Mol. Plant.

[48] Zhang L., Chen F., Zhang G.Q., Zhang Y.Q., Niu S., Xiong J.-S., Lin Z., Cheng Z.M., Liu Z.J. (2016). Origin and mechanism of crassulacean acid metabolism in orchids as implied by comparative transcriptomics and genomics of the carbon fixation pathway. Plant J..

[49] Motomura H., Yukawa T., Ueno O., Kagawa A. (2008). The occurrence of crassulacean acid metabolism in *Cymbidium* (Orchidaceae) and its ecological and evolutionary implications. J. Plant Res..

[50] Li M.H., Liu D.K., Zhang G.Q., Deng H., Tu X.D., Wang Y., Lan S.R., Liu Z.J. (2019). A perspective on crassulacean acid metabolism photosynthesis evolution of orchids on different continents: *Dendrobium* as a case study. J. Exp. Bot..

[51] Torres-Morales G., Lasso E., Silvera K., Turner B.L., Winter K. (2020). Occurrence of crassulacean acid metabolism in Colombian orchids determined by leaf carbon isotope ratios. Bot. J. Linn. Soc..

[52] Zhao Y., Guo A., Wang Y., Hua J. (2019). Evolution of *PEPC* gene family in *Gossypium* reveals functional diversification and *GhPEPC* genes responding to abiotic stresses. Gene.

[53] Ting M.K.Y., She Y.M., Plaxton W.C. (2017). Transcript profiling indicates a widespread role for bacterial-type phosphoenolpyruvate carboxylase in malate-accumulating sink tissues. J. Exp. Bot..

[54] Zhang D., Lan S., Yin W.L., Liu Z.J. (2022). Genome-wide identification and expression pattern analysis of *KNOX* gene family in Orchidaceae. Front. Plant Sci..

[55] Jo B.S., Choi S.S. (2015). Introns, the functional benefits of introns in genomes. Genomics inform..

[56] Hernandez-Garcia C.M., Finer J.J. (2014). Identification and validation of promoters and *cis*-acting regulatory elements. Plant Sci..

[57] Flagel L.E., Wendel J.F. (2009). Gene duplication and evolutionary novelty in plants. New Phytol..

[58] Fay J.C., Wu C.I. (2003). Sequence divergence; functional constraint; and selection in protein evolution. Annu. Rev. Genomics Hum. Genet..

[59] Cvijović I., Good B.H., Desai M.M. (2018). The effect of strong purifying selection on genetic diversity. Genetics.

[60] Wendel J.F., Lisch D., Hu G., Mason A.S. (2018). The long and short of doubling down, polyploidy; epigenetics; and the temporal dynamics of genome fractionation. Curr. Opin. Genet. Dev..

[61] Hsiao Y.Y., Fu C.H., Ho S.Y., Li C.I., Chen Y.Y., Wu W.L., Wang J.S., Zhang D.Y., Hu W.Q., Yu X. (2021). OrchidBase 4.0: A database for orchid genomics and molecular biology. BMC Plant Biol..

[62] Chao Y.-T., Yen S.-H., Yeh J.-H., Chen W.-C., Shih M.-C. (2017). Orchidstra 2.0—A Transcriptomics Resource for the Orchid Family. Plant Cell Physiol..

[63] Chen C., Chen H., Zhang Y., Thomas H.R., Frank M.H., He Y., Xia R. (2020). TBtools, an integrative toolkit developed for interactive analyses of big biological data. Mol. Plant.

[64] Bailey T.L., Johnson J., Grant C.E., Noble W.S. (2015). The MEME suite. Nucleic Acids Res..

[65] Gasteiger E., Hoogland C., Gattiker A., Duvaud S.E., Wilkins M.R., Appel R.D., Bairoch A. (2005). Protein Identification and Analysis Tools on the ExPASy Server.

[66] Chou K.C., Shen H.B. (2010). Plant-mPLoc, a top-down strategy to augment the power for predicting plant protein subcellular localization. PLoS ONE.

[67] Kumar S., Stecher G., Tamura K. (2016). MEGA7: Molecular evolutionary genetics analysis version 7.0 for bigger datasets. Mol. Biol. Evol..

[68] Letunic I., Bork P. (2007). Interactive Tree Of Life (ITOL): An online tool for phylogenetic tree display and annotation. Bioinformatics.

[69] Lescot M., Déhais P., Thijs G., Marchal K., Moreau Y., Van de Peer Y., Rouzé P., Rombauts S. (2002). PlantCARE, a database of plant *cis*-acting regulatory elements and a portal to tools for in silico analysis of promoter sequences. Nucleic Acids Res..

[70] Hurst L.D. (2002). The *Ka/Ks* ratio: Diagnosing the form of sequence evolution. Trends Genet..

